# A cost-effectiveness analysis of early detection and bundled treatment of postpartum hemorrhage alongside the E-MOTIVE trial

**DOI:** 10.1038/s41591-024-03069-5

**Published:** 2024-06-06

**Authors:** Eleanor V. Williams, Ilias Goranitis, Raymond Oppong, Samuel J. Perry, Adam J. Devall, James T. Martin, Kristie-Marie Mammoliti, Leanne E. Beeson, Kulandaipalayam N. Sindhu, Hadiza Galadanci, Fadhlun Alwy Al‑beity, Zahida Qureshi, G. Justus Hofmeyr, Neil Moran, Sue Fawcus, Sibongile Mandondo, Lee Middleton, Karla Hemming, Olufemi T. Oladapo, Ioannis D. Gallos, Arri Coomarasamy, Tracy E. Roberts

**Affiliations:** 1 College of Medical and Dental Sciences, University of Birmingham, Birmingham, UK; 2 Melbourne School of Population and Global Health, University of Melbourne, Melbourne, Victoria Australia; 3 African Center of Excellence for Population Health and Policy, College of Health Sciences, Bayero University, Kano, Nigeria; 4 Department of Obstetrics and Gynecology, Muhimbili University of Health and Allied Sciences, Dar es Salaam, Tanzania; 5 Department of Obstetrics and Gynecology, University of Nairobi, Nairobi, Kenya; 6 Effective Care Research Unit, University of the Witwatersrand, Johannesburg, South Africa; 7 Department of Obstetrics and Gynecology, University of Botswana, Gaborone, Botswana; 8 KwaZulu-Natal Department of Health, Pietermaritzburg, South Africa; 9 Department of Obstetrics and Gynaecology, University of Cape Town, Cape Town, South Africa; 10 Eastern Cape Department of Health, Bhisho, South Africa; 11 Department of Obstetrics and Gynaecology, Walter Sisulu University, Mthatha, South Africa; 12 UNDP/UNFPA/UNICEF/WHO/World Bank Special Programme of Research, Development and Research Training in Human Reproduction, Department of Sexual and Reproductive Health and Research, World Health Organization, Geneva, Switzerland

**Keywords:** Health care economics, Health policy

## Abstract

Timely detection and treatment of postpartum hemorrhage (PPH) are crucial to prevent complications or death. A calibrated blood-collection drape can help provide objective, accurate and early diagnosis of PPH, and a treatment bundle can address delays or inconsistencies in the use of effective interventions. Here we conducted an economic evaluation alongside the E-MOTIVE trial, an international, parallel cluster-randomized trial with a baseline control phase involving 210,132 women undergoing vaginal delivery across 78 secondary-level hospitals in Kenya, Nigeria, South Africa and Tanzania. We aimed to assess the cost-effectiveness of the E-MOTIVE intervention, which included a calibrated blood-collection drape for early detection of PPH and a bundle of first-response treatments (uterine massage, oxytocic drugs, tranexamic acid, intravenous fluids, examination and escalation), compared with usual care. We used multilevel modeling to estimate incremental cost-effectiveness ratios from the perspective of the public healthcare system for outcomes of cost per severe PPH (blood loss ≥1,000 ml) avoided and cost per disability-adjusted life-year averted. Our findings suggest that the use of a calibrated blood-collection drape for early detection of PPH and bundled first-response treatment is cost-effective and should be perceived by decision-makers as a worthwhile use of healthcare budgets. ClinicalTrials.gov identifier: NCT04341662.

## Abstract (web summary)

An economic evaluation of the E-MOTIVE intervention for postpartum hemorrhage (PPH) compared with usual care in 210,132 women, carried out from a healthcare system perspective, uncovered the cost per case of severe PPH prevented and cost per disability-adjusted life-year averted.

## Main

Postpartum hemorrhage (PPH), defined as blood loss ≥500 ml from the genital tract after childbirth, is the leading cause of maternal death worldwide, accounting for approximately 27% of maternal deaths^1,2^. PPH is a major concern in low- and middle-income countries (LMICs), where PPH-associated mortality is disproportionately high^3^. PPH is associated with considerable economic burden: recent estimates from a study conducted in Kenya, India, Nigeria and Uganda suggest the costs of direct hospital care for patients with PPH can be up to 2.8 times higher than for a birth without PPH^4^. In addition, the immediate and long-term economic consequences of maternal mortality incurred by households can be substantial^5–7^.

The World Health Organization (WHO) has published and updated several evidence-informed recommendations for the prevention and treatment of PPH^8,9^. However, adherence to these recommendations in many low-resource settings is limited by numerous challenges. First, PPH is often undetected or detected late; consequently, life-saving treatment is not promptly initiated. The current usual practice of blood-loss assessment is visual estimation, which is widely recognized as inaccurate and typically leads to underestimation of blood loss^10^. An additional challenge is delayed or inconsistent use of effective interventions for the management of PPH. Treatments for PPH are often administered sequentially; healthcare providers wait to observe the effects of one intervention before administering another intervention^11^. However, PPH is a time-critical condition, and such delays can result in loss of life. Some cost-effective interventions may not be used at all. Evidence from hospitals in Kenya, Nigeria, South Africa and Tanzania showed that tranexamic acid (TXA), a medication used to prevent the breakdown of blood clots, was administered late and mostly as a last resort for patients requiring surgery due to PPH^12^. Furthermore, despite the availability of clear recommendations regarding PPH and their wide dissemination, uptake at the point of care remains low^13^. An underpinning factor to some of the challenges relates to limited resources; therefore, it is imperative to evaluate the resource implications of new interventions for managing PPH.

**Fig. 1:**
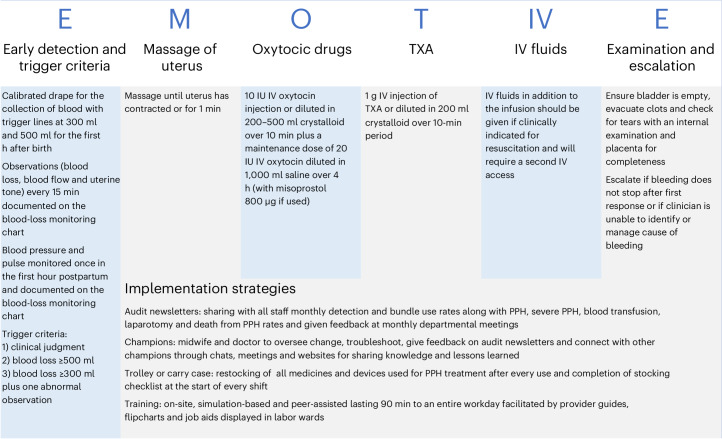
Summary of the E-MOTIVE intervention. The E-MOTIVE intervention included a calibrated blood-collection drape for early detection of PPH and a bundle of first-response treatments (uterine massage, oxytocic drugs, TXA, IV fluids, examination and escalation), supported by an implementation strategy.

To address these challenges, the cluster-randomized E-MOTIVE trial was designed to assess a multicomponent intervention for detection and treatment of PPH in patients having vaginal delivery. The E-MOTIVE intervention consisted of a calibrated blood-collection drape—a sterile fold-out sheet placed on the delivery bed enabling blood to be swept into a pouch with measurement lines indicating warning and action points—for early detection of PPH, and the WHO-proposed first-response bundle, which included uterine massage, oxytocic drugs, TXA, intravenous (IV) fluids and a process for examination and escalation (Fig. 1). The clinical effectiveness of the E-MOTIVE intervention has already been reported^14^. Evidence from the trial supported WHO recommendations for both routine objective measurement of postpartum blood loss for vaginal births, and a standardized and timely approach for managing PPH, comprising objective assessment of blood loss and the bundle, supported by an implementation strategy, for all vaginal births. In this Article, we report the economic evaluation conducted alongside the E-MOTIVE trial, an integral component of the E-MOTIVE project, which aimed to assess the cost-effectiveness of the E-MOTIVE intervention compared with usual care. The economic evaluation, which was carried out from a healthcare system perspective, was based on the outcomes of cost per case of severe PPH prevented (blood loss, ≥1,000 ml) and cost per disability-adjusted life-year (DALY) averted.

## Results

A total of 104 secondary-level hospitals were assessed for eligibility for the E-MOTIVE trial. Fourteen were excluded due to prior implementation of an early-detection protocol or treatment bundle for PPH. Ninety hospitals in Kenya, Nigeria, Pakistan, South Africa and Tanzania entered the baseline phase. The independent data monitoring committee recommended completing the trial before randomizing hospitals in Pakistan, as the required sample size was achieved in the other four countries. Two hospitals in Kenya were excluded before randomization as they were unable to carry out source-data verification.

Eighty hospitals in Kenya, Nigeria, South Africa and Tanzania underwent randomization at a 1:1 ratio to receive the E-MOTIVE intervention or continue providing usual care. Two hospitals in Tanzania, one in each group, did not receive the assigned intervention due to participation in a conflicting program. Following randomization, a 2-month transition was implemented to allow hospitals in the E-MOTIVE group to adapt clinical practices for intervention delivery. Data collected during this phase did not contribute to the analysis.

**Fig. 2:**
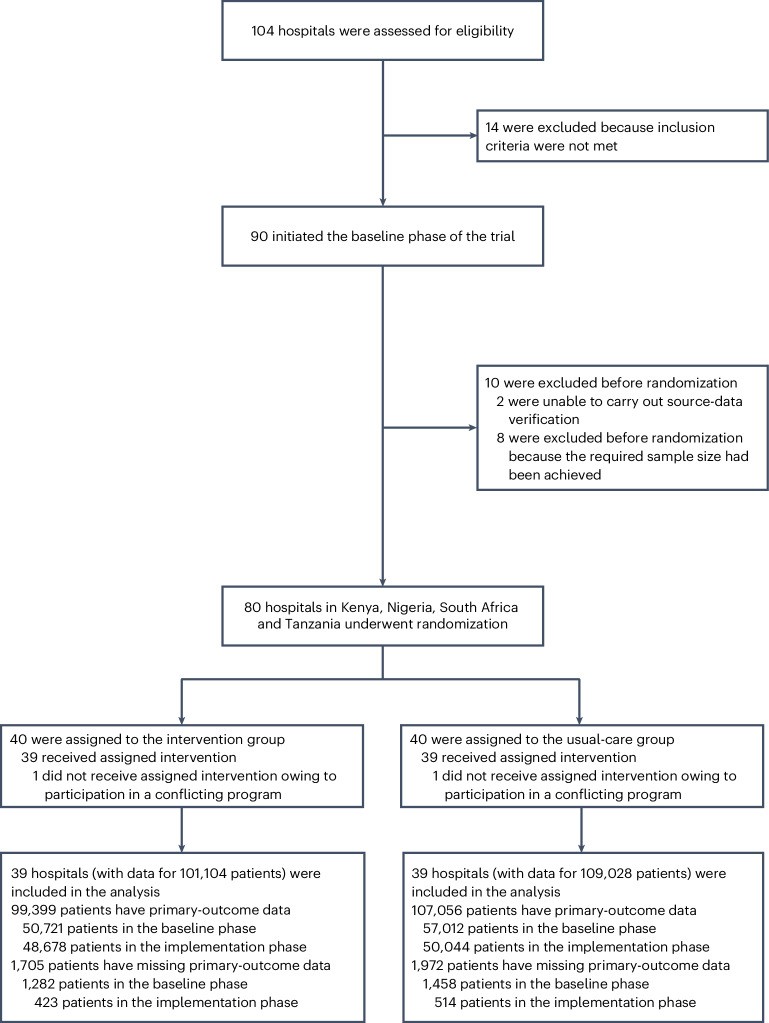
Randomization of hospitals in the E-MOTIVE trial. All participating hospitals entered a 7-month baseline period in which they provided usual care for patients having vaginal delivery. After the baseline phase, hospitals were randomly assigned in a 1:1 ratio to receive the E-MOTIVE intervention or to continue providing usual care. Eighty hospitals across Kenya, Nigeria, South Africa and Tanzania underwent randomization. Due to participating in a conflicting program, two hospitals in Tanzania did not receive the assigned intervention. Data for analysis were therefore available from 78 hospitals, with a total of 210,132 patients. Source-verified blood loss data for analysis were available for 206,455 patients.

Data for analysis were obtained from 78 secondary-level hospitals (from 14 in Kenya, 38 in Nigeria, 14 in South Africa and 12 in Tanzania), with a total of 210,132 patients (110,473 in the baseline phase and 99,659 in the implementation phase) giving birth vaginally in the hospitals between 2 August 2021 and 3 March 2023. Source-verified data regarding blood loss were available for 206,455 patients (107,733 in the baseline phase and 98,722 in the implementation phase; 98% follow-up) (Fig. 2). The clinical findings of the E-MOTIVE trial have been published in full elsewhere^14^.

Severe PPH occurred in 786 of 48,678 patients (1.6%) in the E-MOTIVE group and in 2129 of 50,043 (4.3%) in the usual-care group (adjusted risk difference −2.6%, 95% confidence interval (CI) −3.1% to −2.1%; Table 1). In the E-MOTIVE group, the mean DALYs per patient was 0.00767 (standard deviation (s.d.) 0.394), and in the usual-care group, the mean DALYs per patient was 0.01158 (s.d. 0.454). The adjusted DALY difference between E-MOTIVE and usual care per patient was −0.00266 (95% CI −0.00814 to 0.00287; Table 1).

**Table 1:** Mean per-patient total costs and DALYs, risk of severe PPH and ICERs

	E-MOTIVE (*N* = 48,678)	Usual care (*N* = 50,044)	Adjusted difference^b^(95% CIs^c^)	ICER (2022 USD)
**Mean per-patient total cost (2022 USD)**	45.14 (107.93)	43.19 (126.84)	0.30 (−2.31 to 2.78)	
**Mean per-patient DALYs**	0.00767 (0.394)	0.01158 (0.454)	−0.00266 (−0.00814 to 0.00287)	**113.91**
**Severe PPH**^a^	786 (1.6)	2,129 (4.3)	−2.6 (−3.1 to −2.1)	**11.83**

Values are mean (s.d.) or number (percentage). ^a^Adjusted difference between severe PPH risks is presented in percentage points, and differences between mean values are presented in the unit of the values.

^b^Adjusted for number of vaginal births per hospital, time period, country, the proportion of patients with a clinical primary-outcome event at each hospital and the quality of oxytocin at each hospital during the baseline phase and for clustering using random cluster and cluster-by-period effects. Baseline data before implementation of the intervention (107,733 patients in 78 clusters) for the intervention and usual-care groups are as follows: for mean total cost (USD), 45.43 (134.05) in the E-MOTIVE group and 42.05 (145.37) in the usual-care group; for mean DALYs, 0.01037 (0.427) in the E-MOTIVE group and 0.01314 (0.490) in the usual-care group; for severe PPH, 1,920/50,720 (3.8) in the E-MOTIVE group and 2,535/57,010 (4.4) in the usual-care group.

^c^For total costs and DALYs CIs were constructed using nonparametric permutation tests, by finding the upper and lower boundaries of the intervention effect that would lead to a two-sided *P* value less than the 5% level (1,000 replications).

**Table 2:** Deterministic sensitivity analyses varying calibrated blood-collection drape device cost

Sensitivity analysis	Adjusted mean per-patient total cost difference (2022 USD)^a^	Adjusted mean per-patient DALY difference	ICER (2022 USD)
Drape cost 1 USD	−0.01 (−2.61 to 2.48)	0.00266 (−0.00814 to 0.00287)	**Dominant**^b^
Drape cost 0.75 USD	−0.30 (−2.91 to 2.18)	0.00266 (−0.00814 to 0.00287)	**Dominant**^b^
Drape cost 0.50 USD	−0.61 (−3.22 to 1.87)	0.00266 (−0.00814 to 0.00287)	**Dominant**^b^

Device costs of calibrated drapes are reported before adjustments to 2022 USD and for shipping, handling and internal distribution.

^a^Adjusted for number of vaginal births per hospital, time period, country, the proportion of patients with a clinical primary-outcome event at each hospital and the quality of oxytocin at each hospital during the baseline phase and for clustering using random cluster and cluster-by-period effects.

^b^Dominance is based on point estimate only.

The resource utilization per group is presented in Supplementary Table 1. Notably, administration of oxytocin, TXA and IV fluids—three core elements of the MOTIVE first-response bundle—was more common in the E-MOTIVE group despite lower rates of PPH (8.5% compared with 16.7% in the usual-care group). This can be explained by the improved detection of PPH facilitated by the use of a calibrated blood-collection drape and consequent triggering of the bundle. The usual-care group experienced higher numbers of blood transfusions, marginally longer hospitalization and greater need for additional treatment interventions. Also, notably more severe PPH cases in the usual-care group necessitated additional time for physician attendance.

**Fig. 3:**
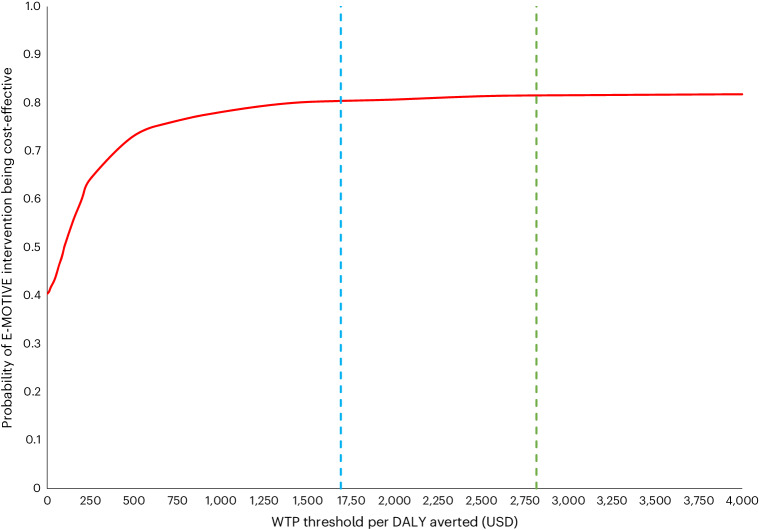
Cost-effectiveness acceptability curve indicating the probability of the E-MOTIVE intervention being cost-effective across different WTP thresholds for a DALY averted. The dashed lines show the expected WTP for a DALY averted, as estimated from WHO recommendations (green) and Woods and colleagues (blue).

Disaggregated mean per-patient costs are presented in Supplementary Table 2. The total unadjusted mean per patient cost was 45.15 USD (s.d. 107.93) in the E-MOTIVE group and 43.19 USD (s.d. 126.84) in the usual-care group (Table 1). The adjusted total cost difference was 0.30 USD (95% CI −2.31 to 2.78; Table 1). The estimated incremental cost-effectiveness ratios (ICERs) (Table 1) are therefore 11.83 USD per case of severe PPH averted and 113.91 USD per DALY averted. The ICER in terms of DALYs is below both the weighted gross domestic product (GDP)-based threshold (2,816 USD) and opportunity-cost based threshold (1,690 USD) (Extended Data Table 1), suggesting the E-MOTIVE intervention is cost-effective. Figure 3 shows the probability of the E-MOTIVE intervention being cost-effective compared with usual care across a range of willingness-to-pay (WTP) thresholds per DALY averted. For thresholds of WTP per DALY averted greater than approximately 1,500 USD, there is >80% probability that the E-MOTIVE intervention is cost-effective (Fig. 3).

### Sensitivity analyses

If the device cost of the calibrated drape is reduced to 1 USD (2023 prices), the E-MOTIVE intervention becomes comparable in cost to usual care, while being more effective (Table 2). Further reductions in the cost of the calibrated drape could potentially result in cost savings. Additional sensitivity analyses to explore the impact of the costing assumptions and the use of multiple imputation (Supplementary Tables 3–5) made no substantial difference to the base-case results; the E-MOTIVE intervention remained cost-effective.

### Country-level analyses

The mean per-patient total costs, DALYs and ICERs from the country-level analyses are presented in Extended Data Table 2. These were estimated using fully pooled, one-country costing models. Briefly, the E-MOTIVE intervention was judged to be cost-effective for each participating country when the ICERs were compared against both country-specific GDP-based WTP thresholds and opportunity-cost-based WTP thresholds (Extended Data Table 1). In South Africa, where the cost of calibrated drapes was lower relative to other resources, the E-MOTIVE intervention was estimated to be less expensive than usual care and, therefore, the dominant intervention based on the point estimates. Accordingly, exploratory analyses (see Supplementary Information, p. 7–11) suggest the budget impact of delivering the E-MOTIVE intervention in these countries would be insubstantial.

## Discussion

This study assessed the cost-effectiveness of early detection of PPH using a calibrated drape and treatment using the WHO first-response treatment bundle, which included uterine massage, oxytocic drugs, TXA, IV fluids and a process for examination and escalation, compared with usual care. The findings suggest that early detection of PPH using a calibrated blood-loss collection drape and treatment with the WHO first-response bundle is cost-effective compared with usual care. Our sensitivity analysis suggested that for WTP values above 1,500 USD per DALY averted there is more than an 80% probability of the E-MOTIVE intervention being cost-effective. Furthermore, deterministic sensitivity analyses showed that potential reductions in the cost of the calibrated blood-collection drape could lead to cost savings, substantially improving the affordability of the E-MOTIVE intervention.

Although a formal quantification of resource use relating to the implementation strategies used to support the E-MOTIVE intervention was not conducted as part of the trial, emerging data suggest the cost of implementation can be effectively absorbed into the existing healthcare system. The post-trial implementation pivot in the four countries indicates that implementing E-MOTIVE does not necessitate additional staffing, and on-site training can be conducted with negligible cost implications. Furthermore, costs related to PPH trolleys or carry cases are minimal and nonrecurrent, while the utilization of audit and feedback solutions and champions does not require additional resources (E-MOTIVE implementation pivot team, personal communication).

The study benefited from a large sample size recruited from 78 hospitals across four countries, broad inclusion criteria to capture all patients with vaginal births in the trial hospitals, and a wide range of primary data. However, the study is not without limitations. Although the analysis considered a range of costs for calibrated blood-collection drapes to account for potential price variations due to increased production, the cost-effectiveness implications of emerging sustainable and climate-friendly alternative devices could not feasibly be assessed^15^. Also, owing to the pragmatic design of the trial, extensive bottom-up costing of all resource items was not conducted. This naturally increases the uncertainty around the unit cost estimates used in the analysis. However, when feasible, cost estimates were obtained from established sources and other secondary sources based on bottom-up costing. Some assumptions were required to estimate country-specific unit costs when these were not available. All assumptions were agreed upon before any analysis was undertaken, and sensitivity analyses exploring their importance found that they did not substantially impact the cost-effectiveness results.

Furthermore, PPH and associated maternal mortality can involve considerable economic costs to patients, their families and wider society^5–7^. Owing to the pragmatic design of the trial, these costs were not captured. Given that there were fewer cases of severe PPH and less severe PPH in the E-MOTIVE group, and maternal deaths from bleeding, though rare, were in the same direction, it is likely that an analysis from the societal perspective—which considers medical and nonmedical costs not directly linked to the intervention—would produce even more favorable cost-effectiveness estimates for the E-MOTIVE intervention.

In addition, this analysis was conducted alongside a large international, cluster-randomized trial with a baseline control phase that presents complexities with respect to data analysis; for example, randomization took place at the cluster level, but outcomes were measured at the level of the individual. This was addressed using methods to account for the hierarchical nature of the data, and the analysis was adjusted for imbalances in outcomes during the baseline phase across trial groups. In addition, due to the substantial loss of power that would be experienced by analyzing countries in isolation, country-specific cost-effectiveness analyses were not conducted. However, we assessed cost-effectiveness from the perspective of each participating country based on whole trial data. To this end, we conducted fully pooled, one-country costing cost-utility analyses (CUAs) in which clinical data from all participating countries were pooled, and country-specific unit costs and life-expectancy data were applied to all patients in the trial. Although not fully country specific, we believe these estimates provide useful indicative information on cost-effectiveness for decision-makers given the widespread occurrence of visual blood loss estimation, and delayed and inconsistent use of effective PPH interventions, such as TXA, across countries. However, these estimates should be interpreted with caution.

Finally, this analysis does not quantify the potential health equity impacts associated with delivering the E-MOTIVE intervention—information likely to be important to decision-makers. The methods of conventional cost-effectiveness analysis (CEA) focus on efficiency, that is, maximizing population health gain from available resources, rather than reducing health inequities. Although frameworks to robustly incorporate equity concerns into CEA are emerging, the substantial data requirements to conduct such an analysis were not feasible for the present analysis.

In summary, our findings suggest that early detection of PPH and bundled treatment for PPH is cost-effective. Therefore, provision of calibrated blood-collection drapes and use of bundled first-response treatment can be considered a worthwhile use of constrained healthcare budgets, and every effort should be made to adhere to the WHO recommendations.

## Methods

### Study design

The E-MOTIVE trial was an international, parallel cluster-randomized trial that included a baseline control phase^14^. A cluster design was required as the E-MOTIVE intervention was delivered at the hospital level, targeting health care providers. Between August and October 2021, all participating hospitals entered a 7-month baseline period during which they provided usual care for PPH in patients having vaginal delivery. Following this 7-month baseline period, hospitals were randomly assigned, in a 1:1 ratio, to continue providing usual care or to receive the E-MOTIVE intervention for 7 months, with a 2-month ‘transition phase’ to allow hospitals to adapt clinical practices for intervention delivery.

A minimization algorithm generated by an independent statistician was used to ensure balance between the intervention hospitals and usual-care hospitals within each country for key prognostic variables, including the number of vaginal births per hospital, the prevalence of primary-outcome events (for the clinical analysis) during the baseline, the quality of oxytocin and the number of hospitals per country.

### Participants

We included secondary-level hospitals in Kenya, Nigeria, South Africa and Tanzania. Hospitals in Pakistan were initially included in the baseline phase but could not be included in the randomization process (Fig. 2). Hospitals were eligible for inclusion if they were geographically and administratively distinct from each other, had between 1,000 and 5,000 vaginal births per year, and were able to provide comprehensive obstetrical care with the ability to perform surgery for PPH. Hospitals were excluded if they had already implemented a treatment bundle for PPH. Written permission was granted by each participating hospital for clinical staff to extract anonymized clinical-outcome data for each vaginal birth.

### Intervention

The E-MOTIVE intervention consisted of a blood-collection drape, with calibrated lines to measure blood-loss volume, for early detection of PPH and the WHO-proposed first-response treatment bundle, which included uterine massage, oxytocic drugs, TXA, IV fluids and a process for examination and escalation (Fig. 1). Detailed information on the E-MOTIVE intervention is published elsewhere^14^.

In usual care, blood loss was estimated visually, with healthcare providers relying on their perceptions to subjectively assess the volume of blood lost. First-response treatment for PPH typically consisted of some or all of the components of the WHO-proposed first-response bundle. These were typically administered sequentially, with oxytocic drugs given as first-line treatment and TXA reserved for refractory bleeding. Established dosage regimens for usual care were applied, consistent with the E-MOTIVE group (Fig. 1).

Noncalibrated drapes, without warning or action lines, were used in the usual-care group hospitals to quantify blood loss for the purpose of the trial.

### Effectiveness outcomes

We estimated cost-effectiveness based on outcomes of severe PPH prevented and DALYs averted.

#### Severe PPH prevented

Severe PPH, defined as blood loss of at least 1,000 ml, was measured at 1 h and, if there was continued bleeding, for up to 2 h postpartum. Blood loss was objectively measured with the use of a blood-collection drape. Calibrated drapes were used in the hospitals in the E-MOTIVE group to enable early and accurate diagnosis of PPH and to obtain data on blood loss. Noncalibrated drapes were used in the hospitals in the usual-care group to obtain data on blood loss. Data on blood loss were source-verified by capturing a photograph of the drape with collected blood inside it, on a digital weighing scale, with the weight visible in the photograph. Only data that had been source-verified were used in the analysis.

This outcome differs from the primary outcome in the E-MOTIVE clinical analysis, which was a composite of severe PPH, laparotomy for bleeding or maternal death from bleeding. Given that composite outcomes are generally inadequate for economic evaluation due to varying component importance, disaggregation is recommended^16^. However, the infrequency of laparotomies and maternal deaths from bleeding in the trial limited a meaningful cost-effectiveness assessment based on these outcomes.

#### DALYs averted

The DALY is a composite summary measure of disease burden that accounts for both mortality and nonfatal health consequences and is the preferred metric for economic evaluations to support resource allocation decisions in LMICs^17^. DALYs were estimated on the basis of nonfatal PPH events and maternal death from bleeding for both arms of the trial.

For nonfatal PPH events, years lived with disability were estimated on the basis of the magnitude of the disability and its duration. Disability weights for severe PPH (0.324 (≥1,000 ml blood lost)) and less severe PPH (0.114 (<1,000 ml blood lost)) were drawn from the Global Burden of Disease study^18^. The duration of disability due to PPH (both severe and less severe) was considered to last for a postpartum period of 6 weeks. Given that the trigger criterion of the E-MOTIVE intervention imposes a benefit on less-severe PPH, it was imperative to include disability for less-severe PPH to ensure relevant effects were captured.

Years of life lost for premature death due to bleeding were calculated using life expectancy of country-specific female populations drawn from Global Burden of Disease abridged life tables^19^. Years of life lost were calculated using a discount rate of 3%, as recommended for economic evaluations in global health^17^.

### Resource use and costs

Resource use information was collected prospectively via electronic case report forms and recorded in REDCap (version 10.9.0–13.3.2). Information was collected from the perspective of the healthcare system for calibrated drapes, uterotonic drugs, TXA, IV fluids, duration of hospitalization, intensive care unit (ICU) admission, transfer to a higher-level facility, blood transfusions, postpartum laparotomy, hysterectomy, nonpneumatic anti-shock garments, uterine balloon tamponades and bimanual compression. When necessary, data from an observational study conducted alongside the E-MOTIVE trial and expert clinical opinion from within the research study team supplemented case report form information.

Extended Data Table 3 presents the unit costs used in the analysis. Calibrated blood-collection drape costs were obtained from Excellent Fixable Drapes in India, the manufacturer and supplier of the drapes used in the E-MOTIVE trial. We considered the price at which the drapes are currently being procured, 1.25 USD, in our base-case analysis. Costs of oxytocic drugs and TXA were obtained from a recent publication by the United States Agency for International Development Global Health Supply Chain Program^20^. Uterotonic drug costs were sourced from the United Nations Populations Fund Product Catalogue, while the TXA costs reported were the United States Agency for International Development wholesale prices. We obtained costs of IV fluids from the International Medical Product Price Guide, a recommended source of medication costs in LMIC settings^21^. An adjustment of 25% was used to account for shipping and handling charges, as well as internal distribution of traded goods^22^.

Country-specific unit cost estimates for non-ICU hospitalization in secondary-level hospitals were obtained from the WHO-CHOICE initiative^23,24^. Country-specific personnel costs were obtained from publicly available records regarding health sector pay, and personal communication with E-MOTIVE country trial management groups^25^; costs from the latter were based on local government salaries. Conservative estimates of the lowest-grade doctor who could attend a case of severe PPH were used. We used other secondary sources to estimate the cost of blood transfusions, additional treatment interventions, transfer to a higher-level facility and ICU admission^4,26–30^.

**Extended Data Fig. 1:**
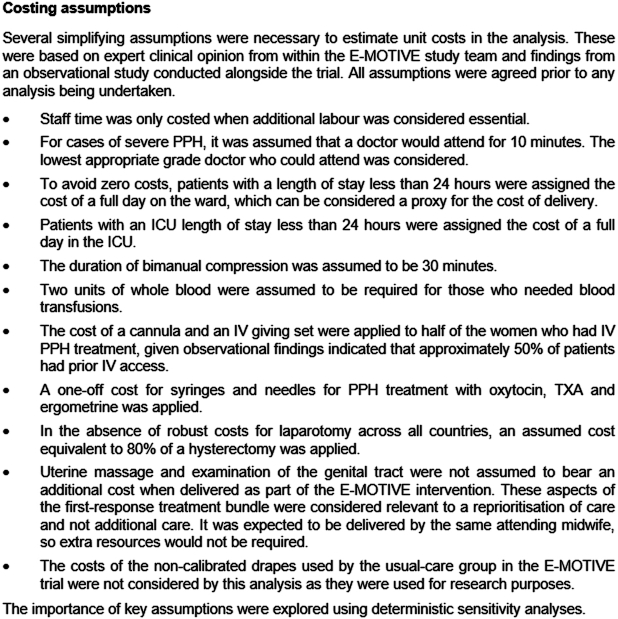
Costing assumptions. Costing assumptions used for economic evaluation.

Due to a lack of cost data for postpartum laparotomy, we assumed a unit cost equivalent to 80% of a hysterectomy, based on expert clinical opinion from within the E-MOTIVE study team. Furthermore, we estimated unit costs for bimanual compression based on personnel requirements and procedure duration, and for uterine balloon tamponades in Kenya, Nigeria and Tanzania, we estimated costs considering materials and labor required for an improvised device. For the base case, we did not apply unit costs to activities perceived as a reprioritization of existing staff time, that is, uterine massage and examination, as we assumed no additional resource was required. Additional details on costing assumptions are provided in Extended Data Fig. 1.

To standardize unit costs across countries where data were unavailable, a market basket approach was used, wherein an index table based on WHO-CHOICE estimates (Extended Data Table 4) was used to indicate the relative mean cost of estimates for inpatient and outpatient health service delivery for each country pair in the study^22–24^. The market basket approach is an established costing method for the development of a complete set of country-specific unit cost data in the economic evaluation of multinational trials^22^. All unit costs were adjusted to 2022 USD using average exchange rates and the average US inflation rate between the price base year used in individual studies and 2022, as recommended when there is a relatively high proportion of imported commodities in economic analyses^31^. Given the short follow-up period of the trial, costs were not discounted.

### Statistical analysis

#### Main analysis

The economic evaluation comprised two main analyses: a CEA based on the outcome of cost per case of severe PPH prevented and a CUA based on the outcome of cost per DALY averted. Both were carried out on an intention-to-treat basis and relied on complete case analysis wherein cases without source-verified blood loss data were excluded.

Following recommendations for the economic evaluation of cluster and multinational trials^32,33^, we used multilevel modeling to estimate the difference in mean costs and outcomes between the E-MOTIVE and usual-care groups. Multilevel modeling accounts for unobserved cluster-specific effects on costs and outcomes and facilitates the estimation of cost-effectiveness across the whole sample^34^. Consistent with the clinical analysis, we fit generalized linear mixed models incorporating a constrained baseline analysis^14^. For severe PPH, we used the binomial family and logit link, in addition to robust standard errors, followed by marginal standardization to estimate risk difference. Differences in mean costs and DALYs were estimated using the Gaussian family and identity link, in combination with nonparametric permutation tests given the inherent skewness of such data^35^. We included fixed effects for allocated exposure to E-MOTIVE, time period, country and covariates used in the randomization method (number of vaginal births per hospital, the proportion of patients with a clinical primary-outcome event at each hospital, and the quality of oxytocin at each hospital during the baseline phase). We adjusted for clustering using random cluster and cluster-by-period effects.

Model estimates of the difference in costs and outcomes were used to derive an incremental cost per case of severe PPH prevented and an incremental cost per DALY averted. For the CUA, we used two thresholds to judge the cost-effectiveness of the E-MOTIVE intervention (Extended Data Table 1): a weighted threshold based on the WHO recommended threshold for a ‘highly cost-effective’ intervention of the countries’ per capita GDP and a weighted threshold based on recently advocated opportunity-cost based thresholds put forward by Woods and colleagues^36–38^, equivalent to 51% GDP per capita for Kenya, Nigeria and Tanzania, and 71% GDP per capita for South Africa.

#### Sensitivity analysis

We conducted sensitivity analyses to quantify the uncertainty relating to key assumptions and sampling variations. To characterize the inherent uncertainty around incremental cost-effectiveness estimates, we used nonparametric clustered bootstrapping with multilevel models to generate 1,000 paired estimates of incremental mean total costs and DALYs. These estimates were used to construct a cost-effectiveness acceptability curve that shows the probability that the E-MOTIVE intervention is cost-effective across a range of WTP threshold values per additional DALY averted^39^. We also conducted deterministic sensitivity analyses on input parameters for the base-case analysis (Supplementary Information, p. 3). This included varying the device cost of the calibrated drapes to 1 USD, 0.75 USD and 0.50 per unit (2023 prices) respectively, considering potential price decreases with expanded production.

Given that only source-verified blood-loss data were used in the main analysis, we conducted a sensitivity analysis using multiple imputation to assess the effect of missing data. Missing data were imputed under the assumption that data were missing at random, with an allowance for clustering. The multiple imputation was performed using chained equations. Differences between the E-MOTIVE and usual-care groups in terms of risk of severe PPH, means costs and mean DALYs from the seven multiply imputed datasets were obtained using multilevel models in the same manner as the main analysis and pooled using Rubin’s rules.

#### Country-level analysis

To provide indicative context for local decision-makers, we estimated the cost-effectiveness of the E-MOTIVE intervention from the perspective of each participating country using four fully pooled, one-country costing CUAs. Clinical outcome and utilization data from all participating countries were pooled, and country-specific unit costs and life-expectancy data were applied to all patients in the trial. The country-level analyses were adjusted analogously to the main analyses. Model estimates of differences in cost and DALYs were used to derive ICERs, which were judged against the country-specific thresholds reported in Extended Data Table 4. We extended these estimates to explore the potential budget impact of implementing the E-MOTIVE intervention (Supplementary Information, p. 7–11).

All analyses were carried out using Stata, version 17.1 (StataCorp).

### Ethical approval

Ethical approval was granted by the University of Birmingham Science, Technology, Engineering and Mathematics (STEM) ethics committee in the UK (ERN_19-1557); the World Health Organization – Human Reproduction Programme (WHO-HRP) (approval for formative phase) in Switzerland; the Kenyatta National Hospital (KNH) – University of Nairobi (UoN) Ethics and Research Committee (KNH-ERC/A/197), the National Commission for Science, Technology and Innovation (NACOSTI) (NACOSTI/P/21/8330), and the Pharmacy and Poisons Board (PPB) in Kenya (PPB/ECCT/20/06/08/2020(122)); the National Health Research Ethics Committee of Nigeria (NHREC) (NHREC/01/01/2007) and National Agency for Food and Drug Administration and Control (NAFDAC) in Nigeria (NAFDAC/DER/VCTD/E-MOTIVE/2022); the University of the Witwatersrand Human Research Ethics Committee (Medical) (M200241), the Eastern Cape Department of Health – Eastern Cape Health Research Committee (EC_202007_014), the KwaZulu-Natal Department of Health – KZN Health Research Committee (KZ_202008_036) and the University of Cape Town – Human Research Ethics Committee in South Africa (091/2020); the Muhimbili University of Health and Allied Sciences (MUHAS) – Senate Research and Publications Committee (DA.282/298/01.C/) and the National Institute for Medical Research in Tanzania (NIMR/HQ/R.8a/Vol. IX/3510). The trial was registered on ClinicalTrials.gov (NCT04341662) and the Pan African Clinical Trials Registry (PACTR202002791391791). All participants provided written informed consent before participation in intervention training.

### Inclusion and ethics statement

Local researchers, including national principal investigators from each participating country, contributed to the E-MOTIVE study design. National principal investigators also led the implementation of the study in their respective countries, supported by a national team of local study coordinators and data managers. Additionally, local research midwives/nurses were also employed at each hospital to facilitate data collection and adherence to study protocols. Moreover, both national principal investigators and local study coordinators are acknowledged as authors of publications arising from the E-MOTIVE study.

This research is locally relevant to each of the participating countries as maternal mortality rates due to PPH are highest in sub-Saharan Africa. Co-design workshops were conducted in each country before implementing the E-MOTIVE intervention, which enabled key local stakeholders to contribute to discussions on adapting implementation strategies to local contexts.

Roles and responsibilities were agreed upon among collaborators ahead of the research. Capacity-building plans for local researchers focused on training research hub staff to conduct a large international, cluster-randomized trial, and on training local research midwives/nurses to facilitate implementation of the E-MOTIVE intervention during client care.

This research would not have been severely restricted or prohibited in the setting of the researchers and does not result in stigmatization, incrimination or discrimination to participants. There is a risk to participants (healthcare providers) if their personal data are not adequately protected. However, the study strictly adhered to applicable data protection regulations in each country, including de-identifying data collected from interviews and surveys before review and conducting on-site monitoring visits to ensure secure storage of participant data.

A central sponsor (University of Birmingham) level risk assessment was put in place during the setup phase of the study. Subsequently, within each country, a separate risk assessment was developed in collaboration with the national coordinating team and finalized before data collection commenced. A central and country-specific monitoring plan and data management plan were also put in place.

Lastly, local and regional research relevant to our study was taken into account in the write-up of this manuscript and the wider E-MOTIVE project.

### Reporting summary

Further information on research design is available in the Nature Portfolio Reporting Summary linked to this article.

## Online content

Any methods, additional references, Nature Portfolio reporting summaries, source data, extended data, supplementary information, acknowledgements, peer review information; details of author contributions and competing interests; and statements of data and code availability are available at 10.1038/s41591-024-03069-5.

### Supplementary information

Supplementary Information Supplementary Tables 1 and 2. List of deterministic sensitivity analysis conducted. Supplementary Tables 3–5. Potential budget impact analysis. Supplementary Tables 6–9.

Reporting Summary

## Figures and Tables

**Extended Data Table 1:**
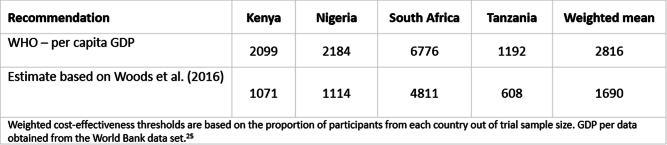
Willingness-to-pay (USD) estimates for a disability-adjusted life-year averted in the participating countries

**Extended Data Table 2:**
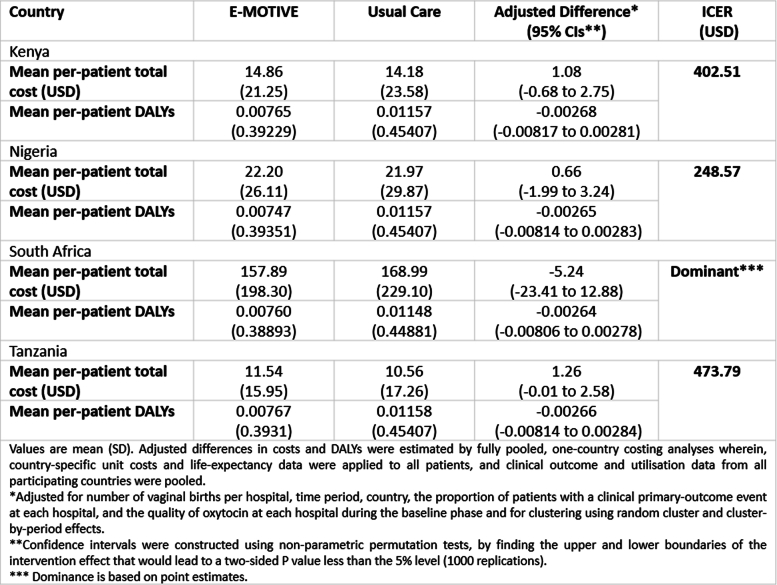
Country-level estimates of mean per-patient total costs (2022 USD), DALYs, and ICERs

**Extended Data Table 3:**
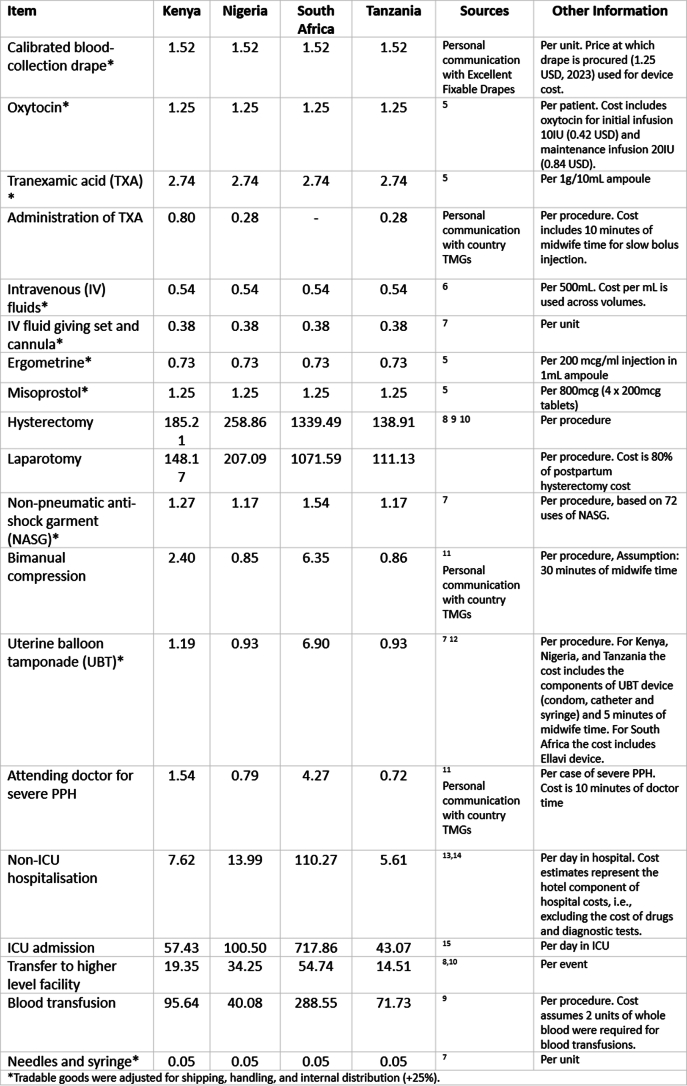
Unit Costs (2022 USD)

**Extended Data Table 4:**
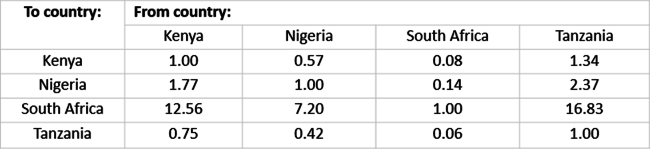
Relative cost indices of participating countries

## Data Availability

Patient data cannot be made publicly available due to privacy concerns. The complete de-identified patient data that support the findings of this study can be obtained from the Chief Investigator of the E-MOTIVE trial, on approval from the E-MOTIVE Trial Data Analysis Sub-Committee. Approval from this committee can be requested by directly contacting the Chief Investigator (a.coomarasamy@bham.ac.uk), with an expected review period of approximately 2–3 months. After approval, researchers will be granted access to perform analyses, ensuring data security and confidentiality, with measures in place to prevent any breach of personal information. Additional data used for the analysis are publicly available and referenced in Methods and Supplementary Information. The parameter values and their sources are reported in Extended Data Table 3 and Supplementary Tables 6–9.

## Code availability

Stata codes are available via GitHub at https://github.com/ewbham/E-MOTIVE.

## References

[1] Kassebaum, N. J. et al. Global, regional, and national levels and causes of maternal mortality during 1990-2013: a systematic analysis for the Global Burden of Disease Study 2013. *Lancet* **384**, 980–1004 (2014). 24797575 10.1016/S0140-6736(14)60696-6 PMC4255481

[2] Sheldon, W. R. et al. Postpartum haemorrhage management, risks, and maternal outcomes: findings from the World Health Organization Multicountry Survey on Maternal and Newborn Health. *BJOG* **121**, 5–13 (2014). 24641530 10.1111/1471-0528.12636

[3] Say, L. et al. Global causes of maternal death: a WHO systematic analysis. *Lancet Glob. Health* **2**, e323–e333 (2014). 25103301 10.1016/S2214-109X(14)70227-X

[4] Theunissen, F. et al. Cost of hospital care of women with postpartum haemorrhage in India, Kenya, Nigeria and Uganda: a financial case for improved prevention. *Reprod. Health* **18**, 18 (2021). 33482858 10.1186/s12978-020-01063-x PMC7821537

[5] Kes, A. et al. The economic burden of maternal mortality on households: evidence from three sub-counties in rural western Kenya. *Reprod. Health* **12**, S3 (2015). 26000953 10.1186/1742-4755-12-S1-S3 PMC4423575

[6] Molla, M., Mitiku, I., Worku, A. & Yamin, A. Impacts of maternal mortality on living children and families: a qualitative study from Butajira, Ethiopia. *Reprod. Health* **12**, S6 (2015). 26001276 10.1186/1742-4755-12-S1-S6 PMC4423766

[7] Bazile, J. et al. Intergenerational impacts of maternal mortality: qualitative findings from rural Malawi. *Reprod. Health* **12**, S1 (2015). 26000733 10.1186/1742-4755-12-S1-S1 PMC4423580

[8] *WHO Recommendations for the Prevention and Treatment of Postpartum Haemorrhage* (World Health Organization, 2012). 23586122

[9] Vogel, J. P., Oladapo, O. T., Dowswell, T. & Gülmezoglu, A. M. Updated WHO recommendation on intravenous tranexamic acid for the treatment of post-partum haemorrhage. *Lancet Glob. Health* **6**, e18–e19 (2018). 29100880 10.1016/S2214-109X(17)30428-X

[10] Hancock, A., Weeks, A. D. & Lavender, D. T. Is accurate and reliable blood loss estimation the ‘crucial step’ in early detection of postpartum haemorrhage: an integrative review of the literature. *BMC Pregnancy Childbirth* **15**, 230 (2015). 26415952 10.1186/s12884-015-0653-6 PMC4587838

[11] Althabe, F. et al. Postpartum hemorrhage care bundles to improve adherence to guidelines: a WHO technical consultation. *Int J. Gynaecol. Obstet.* **148**, 290–299 (2020). 31709527 10.1002/ijgo.13028 PMC7064978

[12] Forbes, G. et al. Factors influencing postpartum haemorrhage detection and management and the implementation of a new postpartum haemorrhage care bundle (E-MOTIVE) in Kenya, Nigeria, and South Africa. *Implement. Sci.* **18**, 1 (2023). 36631821 10.1186/s13012-022-01253-0 PMC9832403

[13] Akter, S. et al. Detection and management of postpartum haemorrhage: qualitative evidence on healthcare providers’ knowledge and practices in Kenya, Nigeria, and South Africa. *Front. Glob. Women’s Health* **3**, 1020163 (2022). 36467287 10.3389/fgwh.2022.1020163 PMC9715762

[14] Gallos, I. et al. Randomized trial of early detection and treatment of postpartum hemorrhage. *N. Engl. J. Med.* **389**, 11–21 (2023). 37158447 10.1056/NEJMoa2303966

[15] Singata-Madliki, M. & Hofmeyr, G. J. A novel, re-usable ‘Safe birth Tray’ for postpartum blood loss monitoring: a preliminary acceptability assessment. *Int. J. Gynaecol. Obstet.* **155**, 553–555 (2021). 34227106 10.1002/ijgo.13817

[16] Ramsey, S. D. et al. Cost-effectiveness analysis alongside clinical trials II-An ISPOR Good Research Practices Task Force report. *Value Health* **18**, 161–172 (2015). 25773551 10.1016/j.jval.2015.02.001

[17] Wilkinson, T. et al. The international decision support initiative reference case for economic evaluation: an aid to thought. *Value Health* **19**, 921–928 (2016). 27987641 10.1016/j.jval.2016.04.015

[18] Global Burden of Disease Collaborative Network. *Global Burden of Disease Study 2019 (GBD 2019) Disability Weights* (Institute for Health Metrics and Evaluation, 2020).

[19] Global Burden of Disease Collaborative Network. *Global Burden of Disease Study 2019 (GBD 2019) Life* Tables 1950–2019 (Institute for Health Metrics and Evaluation, 2020).

[20] *Uses of Medicines for Prevention and Treatment of Post-partum Hemorrhage and Other Obstetric Purposes* (United States Agency for International Development, 2022).

[21] *International Medical Product Price Guide* (Management Sciences for Health, 2015).

[22] Mulligan, J.-A. et al. Unit costs of health care inputs in low and middle income regions. Disease control priorities project working paper. *LSHTM Research Online* http://researchonline.lshtm.ac.uk/12952/ (LSHTM, 2005).

[23] *WHO-CHOICE Estimates of Cost for Inpatient and Outpatient Health Service Delivery* (World Health Organization, 2021).

[24] Stenberg, K., Lauer, J. A., Gkountouras, G., Fitzpatrick, C. & Stanciole, A. Econometric estimation of WHO-CHOICE country-specific costs for inpatient and outpatient health service delivery. *Cost. Eff. Resour. Alloc.* **16**, 11 (2018). 29559855 10.1186/s12962-018-0095-x PMC5858135

[25] *Salary Scales, with Translation Keys, for Employees on Salary Levels 1 to 12 and Those Employees Covered by Occupation Specific Dispensions (OSDs)* (Department of Public Service and Administration, 2023).

[26] *UNICEF Supply Catalogue* Vol. 2023 (UNICEF Supply Division, 2023).

[27] Mvundura, M. et al. Cost-effectiveness of condom uterine balloon tamponade to control severe postpartum hemorrhage in Kenya. *Int J. Gynaecol. Obstet.* **137**, 185–191 (2017). 28190262 10.1002/ijgo.12125

[28] *Uniform Patient Fee Schedule 2022* Vol. 2023 (National Department of Health, 2022).

[29] Theron, G. B. Management of postpartum hemorrhage with free-flow pressure controlled uterine balloon. *Int J. Gynaecol. Obstet.* **142**, 371–373 (2018). 29779208 10.1002/ijgo.12533

[30] Dayananda, K. et al. Selective non-operative management of abdominal stab wounds is a safe and cost effective strategy: a South African experience. *Ann. R. Coll. Surg. Engl.* **99**, 490–496 (2017). 28660819 10.1308/rcsann.2017.0075 PMC5696983

[31] Kumaranayake, L. The real and the nominal? Making inflationary adjustments to cost and other economic data. *Health Policy Plan* **15**, 230–234 (2000). 10837047 10.1093/heapol/15.2.230

[32] Gomes, M. et al. Developing appropriate methods for cost-effectiveness analysis of cluster randomized trials. *Med. Decis. Mak.* **32**, 350–361 (2012). 10.1177/0272989X11418372 PMC3757919 22016450

[33] Manca, A., Sculpher, M. J. & Goeree, R. The analysis of multinational cost-effectiveness data for reimbursement decisions: a critical appraisal of recent methodological developments. *Pharmacoeconomics* **28**, 1079–1096 (2010). 21080734 10.2165/11537760-000000000-00000

[34] Manca, A., Rice, N., Sculpher, M. J. & Briggs, A. H. Assessing generalisability by location in trial-based cost-effectiveness analysis: the use of multilevel models. *Health Econ.* **14**, 471–485 (2005). 15386662 10.1002/hec.914

[35] Thompson, J., Davey, C., Hayes, R., Hargreaves, J. & Fielding, K. swpermute: permutation tests for stepped-wedge cluster-randomised trials. *Stata J.* **19**, 803–819 (2019). 32565746 10.1177/1536867X19893624 PMC7305031

[36] Woods, B., Revill, P., Sculpher, M. & Claxton, K. Country-level cost-effectiveness thresholds: initial estimates and the need for further research. *Value Health* **19**, 929–935 (2016). 27987642 10.1016/j.jval.2016.02.017 PMC5193154

[37] *The World Health Report 2002: Reducing Risks, Promoting Healthy Life* (World Health Organization, 2002). 10.1080/1357628031000116808 14741909

[38] *GDP per Capita (Current US$)* (The World Bank, 2023).

[39] Fenwick, E., Claxton, K. & Sculpher, M. Representing uncertainty: the role of cost-effectiveness acceptability curves. *Health Econ.* **10**, 779–787 (2001). 11747057 10.1002/hec.635

