# Supplementary material for: A cost-effectiveness analysis of early detection and bundled treatment of postpartum hemorrhage alongside the E-MOTIVE trial
Source: Nat Med. 2024 Jun 6;30(8):2343–8. doi: 10.1038/s41591-024-03069-5 (PMC11333277; doi:10.1038/s41591-024-03069-5)
Supplement: Supplementary file 1 — Supplementary Tables 1 and 2. List of deterministic sensitivity analysis conducted. Supplementary Tables 3–5. Potential budget impact analysis. Supplementary Tables 6–9. [file 41591_2024_3069_MOESM1_ESM.pdf]

nature medicine

Supplementary information

https://doi.org/10.1038/s41591-024-03069-5

A cost-effectiveness analysis of early detection and bundled treatment of postpartum hemorrhage alongside the E-MOTIVE trial

In the format provided by the authors and unedited

Supplementary Information

Supplementary Table 1. Resource use per group		
	**E-MOTIVE (N = 48,678)**	**Usual care (N = 50,043)**
Uterine massage	5,762 (11.84)	4,085 (8.16)
Oxytocin use	5,864 (12.05)	4,283 (8.56)
Tranexamic acid use	5,796 (11.91)	1,965 (3.93)
Intravenous fluids use	5,851 (12.02)	4,163 (8.32)
Examination of the genital tract	5,476 (11.25)	3,581 (7.16)
Ergometrine use	124 (0.25)	75 (0.15)
Misoprostol use	2,587 (5.31)	2,705 (5.41)
Laparotomy	1 (0.00)	3 (0.01)
Hysterectomy	11 (0.02)	6 (0.01)
Non-pneumatic anti-shock garment (NASG) use	89 (0.18)	38 (0.08)
Bimanual compression	136 (0.28)	553 (1.11)
Uterine balloon tamponade use	44 (0.09)	57 (0.11)
Blood transfusion	1,063 (2.18)	1,286 (2.57)
Transfer to higher level facility	82 (0.17)	17 (0.03)
Intensive care unit (ICU) admissions	7 (0.01)	28 (0.06)
Duration of hospitalisation (days)*	1.09 (1.78)	1.14 (2.06)
Duration of ICU hospitalisation (days)*	2.00 (2.45)	1.57 (1.53)

Values are number (percentage) or mean (SD)*

Baseline data before implementation of the intervention (107,733 patients in 78 clusters) for the E-MOTIVE and usual-care groups are as follows: *Uterine massage:* E-MOTIVE: 5,074 (10.00), usual care: 4,537 (7.96); *Oxytocin use:* E-MOTIVE: 5,740 (11.32), usual care: 4,063 (8.88); *Tranexamic acid use:* E-MOTIVE: 2,246 (4.43), usual care: 1,221 (2.14); *Intravenous fluid use:* E-MOTIVE: 5,289 (10.43), usual care: 4,458 (7.82); *Examination of the genital tract:* E-MOTIVE: 4,007 (7.90), usual care: 3,786 (6.64); *Ergometrine use:* E-MOTIVE: 131 (0.26), usual care: 162 (0.28); *Misoprostol use:* E-MOTIVE: 3,286 (6.48), usual care: 3,234 (5.67); *Laparotomy:* E-MOTIVE: 3 (0.01), usual care: 3 (0.01); *Hysterectomy:* E-MOTIVE: 7 (0.01), usual care: 8 (0.01); *NASG:* E-MOTIVE: 72 (0.14), usual care: 50 (0.09); *Bimanual compression:* E-MOTIVE: 303 (0.60), usual care: 438 (0.77); *Uterine balloon tamponade:* E-MOTIVE: 23 (0.05), usual care: 56 (0.11); *Blood transfusion:* E-MOTIVE: 1,474 (2.91), usual care: 1,652 (2.90); *Transfer to higher level facility:* E-MOTIVE: 74 (0.15), usual care: 23 (0.04); *ICU admissions:* E-MOTIVE: 13 (0.03), usual care: 39 (0.07); *Duration of hospitalisation (days)**: E-MOTIVE: 1.06 (2.31), usual care: 1.05 (1.79); *Duration of ICU hospitalisation (days)**: E-MOTIVE: 2.00 (1.96), usual care: 2.02 (3.09).

Supplementary Table 2. Mean per-patient costs (2022 USD)				
	**E-MOTIVE (N= 48,678)**	**Usual care (N = 50,043)**	**Adjusted Difference***	**95% CIs****
Calibrated blood-collection drape	1.518 (0.000)	0.000 (0.000)	1.518	-
Oxytocin	0.151 (0.408)	0.111 (0.357)	0.034	0.007 to 0.068
Tranexamic acid use	0.367 (1.00)	0.126 (0.624)	0.252	0.199 to 0.305
Intravenous fluids use	0.216 (0.582)	0.162 (0.512)	0.047	0.007 to 0.087
Misoprostol	0.066 (0.280)	0.068 (0.282)	0.004	-0.019 to 0.026
Ergometrine	0.002 (0.037)	0.001 (0.026)	0.002	0.000 to 0.003
Needles and syringes	0.006 (0.016)	0.005 (0.014)	0.001	0.000 to 0.002
Laparotomy	0.003 (0.672)	0.009 (1.241)	-0.008	-0.019 to 0.005
Hysterectomy	0.117 (10.875)	0.025 (2.333)	0.101	-0.030 to 0.228
Non-pneumatic anti shock garment (NASG)	0.002 (0.051)	0.001 (0.035)	0.001	-0.001 to 0.003
Bimanual compression	0.003 (0.093)	0.013 (0.178)	-0.004	-0.012 to 0.004
Uterine balloon tamponade (UBT)	0.002 (0.076)	0.001 (0.055)	0.000	-0.001 to 0.002
Blood transfusion	1.348 (12.710)	1.899 (16.940)	-0.088	-0.585 to 0.439
Non-ICU hospitalisation	41.175 (104.015)	40.526 (118.889)	-1.718	-3.823 to 0.396
ICU admission	0.037 (3.996)	0.439 (31.238)	0.194	-0.196 to 0.689
Transfer to higher level facility	0.089 (2.2017)	0.011 (0.707)	0.024	-0.001 to 0.047
Severe postpartum haemorrhage (doctor time)	0.034 (0.334)	0.0622 (0.3949)	-0.027	-0.035 to -0.018
**Mean total cost (USD)**	**45.135 (107.932)**	**43.189 (126.844)**	0.302	-2.312 to 2.783

Values are mean (SD).
*Adjusted for number of vaginal births per hospital, time period, country, the proportion of patients with a clinical primary-outcome event at each hospital and the quality of oxytocin at each hospital during the baseline phase and for clustering using random cluster and cluster-by-time effects.
** Confidence intervals were constructed using non-parametric permutation tests, by finding the upper and lower boundaries of the intervention effect that would lead to a two-sided P value less than the 5% level (1000 replications).

List of deterministic sensitivity analyses conducted.

1. Reduced calibrated drape device cost to 1 USD (2023 price).
2. Reduced calibrated drape device cost to 0.75 USD (2023 price).
3. Reduced calibrated drape device cost to 0.50 USD (2023 price).
4. Assigned cost to 2 minutes of midwife time for uterine massage and 5 minutes for examination of the genital tract.
5. Increased the number of units of whole blood required for transfusion to 3 units.
6. Reduced the number of units of whole blood required for transfusion to 1 unit.
7. Increased midwife time for bimanual compression to 45 minutes.
8. Reduced midwife time for bimanual compression to 15 minutes.
9. Reduced doctor time required for attending SPPH to 5 minutes.
10. Reduced the cost of laparotomy to 50% of the cost of hysterectomy.

**Supplementary Table 3. Results of deterministic sensitivity analyses**		
1) Reduced calibrated drape device cost to 1 USD (2023 prices)		
	**Adjusted difference (E-MOTIVE – usual care)* (95% CIs)****	**ICER (2022 USD)**
**Mean per-patient total cost (2022 USD)**	-0.01 (-2.61 to 2.48)	**-**
**Risk of severe PPH**	-2.6 (-3.1 to -2.1)	Dominant
**Mean per-patient DALYs**	-0.00266 (-0.00814 to 0.00287)	Dominant
2) Reduced calibrated drape device cost to 0.75 USD (2023 prices)		
	**Adjusted difference (E-MOTIVE – usual care)* (95% CIs)****	**ICER (2022 USD)**
**Mean per-patient total cost (USD)**	-0.30 (-2.91 to 2.18)	**-**
**Risk of severe PPH**	-2.6 (-3.1 to -2.1)	Dominant
**Mean per-patient DALYs**	-0.00266 (-0.00814 to 0.00287)	Dominant
3) Reduced calibrated drape device cost to 0.50 USD (2023 prices)		
	**Adjusted difference (E-MOTIVE – usual care)* (95% CIs)****	**ICER (2022 USD)**
**Mean per-patient total cost (USD)**	-0.61 (-3.22 to 1.87)	**-**
**Risk of severe PPH**	-2.6 (-3.1 to -2.1)	Dominant
**Mean per-patient DALYs**	-0.00266 (-0.00814 to 0.00287)	Dominant
4) Assigned cost to 2 minutes of midwife time for uterine massage and 5 minutes for examination of the genital tract		
	**Adjusted difference (E-MOTIVE – usual care)* (95% CIs)****	**ICER (2022 USD)**
**Mean per-patient total cost (USD)**	0.37	-
**Risk of severe PPH**	-2.6 (-3.1 to -2.1)	14.39
**Mean per-patient DALYs**	-0.00266 (-0.00814 to 0.00287)	139.26
5) Increased the number of units of whole blood required for transfusion to 3 units		
	**Adjusted difference (E-MOTIVE – usual care)* (95% CIs)****	**ICER (2022 USD)**
**Mean per-patient total cost (USD)**	0.23 (-2.52 to 2.84)	-
**Risk of severe PPH**	-2.6 (-3.1 to -2.1)	8.86
**Mean per-patient DALYs**	-0.00266 (-0.00814 to 0.00287)	85.76

* Adjusted difference between severe PPH risks is presented in percentage points, and differences between mean values are presented in the unit of the values. Adjusted for number of vaginal births per hospital, time period, country, the proportion of patients with a clinical primary-outcome event at each hospital and the quality of oxytocin at each hospital during the baseline phase and for clustering using random cluster and cluster-by-period effects.
** Confidence intervals were constructed using non-parametric permutation tests, by finding the upper and lower boundaries of the intervention effect that would lead to a two-sided P value less than the 5% level (1000 replications).

Supplementary Table 4. Results of deterministic sensitivity analyses (continued)		
6) Reduced the number of units of whole blood required for transfusion to 1 units		
	**Adjusted difference (E-MOTIVE – usual care)* (95% CIs)****	**ICER (2022 USD)**
**Mean per-patient total cost (USD)**	0.38 (-2.50 to 2.74)	-
**Risk of severe PPH**	-2.6 (-3.1 to -2.1)	14.83
**Mean per-patient DALYs**	-0.00266 (-0.00814 to 0.00287)	143.53
7) Increased midwife time for bimanual compression to 45 minutes.		
	**Adjusted difference (E-MOTIVE – usual care)* (95% CIs)****	**ICER (2022 USD)**
**Mean per-patient total cost (USD)**	0.30 (-2.31 to 2.78)	-
**Risk of severe PPH**	-2.6 (-3.1 to -2.1)	11.71
**Mean per-patient DALYs**	-0.00266 (-0.00814 to 0.00287)	113.33
8) Reduced midwife time for bimanual compression to 15 minutes		
	**Adjusted difference (E-MOTIVE – usual care)* (95% CIs)****	**ICER (2022 USD)**
**Mean per-patient total cost (USD)**	0.30 (-2.31 to 2.78)	-
**Risk of severe PPH**	-2.6 (-3.1 to -2.1)	11.83
**Mean per-patient DALYs**	-0.00266 (-0.00814 to 0.00287)	114.45
9) Reduced doctor time required for attending SPPH to 5 minutes		
	**Adjusted difference (E-MOTIVE – usual care)* (95% CIs)****	**ICER (2022 USD)**
**Mean per-patient total cost (USD)**	0.31 (-2.32 to 2.80)	-
**Risk of severe PPH**	-2.6 (-3.1 to -2.1)	12.20
**Mean per-patient DALYs**	-0.00266 (-0.00814 to 0.00287)	118.11
10) Reduced the cost of laparotomy to 50% of the cost of hysterectomy		
	**Adjusted difference (E-MOTIVE – usual care)* (95% CIs)****	**ICER (2022 USD)**
**Mean per-patient total cost (USD)**	0.30 (-2.31 to 2.79)	-
**Severe PPH Risk**	-2.6 (-3.1 to -2.1)	11.88
**Mean per-patient DALYs**	-0.00266 (-0.00814 to 0.00287)	114.84

* Adjusted difference between severe PPH risks is presented in percentage points, and differences between mean values are presented in the unit of the values. Adjusted for number of vaginal births per hospital, time period, country, the proportion of patients with a clinical primary-outcome event at each hospital and the quality of oxytocin at each hospital during the baseline phase and for clustering using random cluster and cluster-by-period effects.

** Confidence intervals were constructed using non-parametric permutation tests, by finding the upper and lower boundaries of the intervention effect that would lead to a two-sided P value less than the 5% level (1000 replications).

Supplementary Table 5. Results of sensitivity analysis using multiple imputation		
	**Adjusted difference (E-MOTIVE – usual care)* (95% CIs)**	**ICER (2022 USD)**
**Mean per-patient total cost (USD)**	0.25 (-2.32 to 2.69)	-
**Mean per-patient DALYs**	-0.00286 (-0.00836 to 0.00264)	88.66
**Risk of severe PPH**	-2.6 (-3.1 to -2.1)	9.87
*Adjusted difference between severe PPH risks is presented in percentage points, and differences between mean values are presented in the unit of the values. Adjusted for number of vaginal births per hospital, time period, country, the proportion of patients with a clinical primary-outcome event at each hospital and the quality of oxytocin at each hospital during the baseline phase and for clustering using random cluster and cluster-by-period effects.		

Budget Impact Analysis

Methods

We conducted a budget impact analysis (BIA) for each participating country, based on country-level cost-effectiveness estimates, to assess the potential implications of introducing the E-MOTIVE intervention on public healthcare systems expenditure over a 5-year period.

We sourced data on the projected annual number of births from the World Population Prospects 2022 by the Population Division of the United Nations Department of Economic and Social Affairs.[1] Data on the proportion of births in public hospitals, and the proportion of vaginal births were obtained from the most recent Demography and Health Survey (DHS) of each participating country.[2-5]

Based on discussions within the E-MOTIVE study team, we assumed a linear growth in implementation of the E-MOTIVE intervention in public hospitals from 10% in Year 1 (2024) to 100% in Year 3 (2026). The initial training required for E-MOTIVE implementation was considered a one-off cost before its integration into standard training practices. We estimated training costs based on staff time required, in alignment with table A1, and included a 10% additive adjustment to account for overhead costs and training materials.

For the E-MOTIVE trial, training was conducted at two levels: training of trainers (ToT) and on-site training. Based on discussion with the E-MOTIVE study team, we assumed that one doctor and two midwives attended ToT for 2.5 days and facilitated the training of 20 midwives at their facility. On-site training, led by 1 trainer for 10 midwives, lasted for 1.5 days. Hospitals in the E-MOTIVE trial had approximately 20 midwives and 2000 vaginal births annually: training costs were apportioned accordingly to determine a per-delivery training cost. These costs were incorporated for all deliveries in Year 1, and subsequent uptakes in Years 2 and 3 respectively. Given the assumption that all public hospitals implemented the E-MOTIVE intervention by Year 3, no training costs were applied for Years 4 and 5.

In sensitivity analyses, we explored the potential budget impact of reducing the device cost of calibrated drapes to 1 USD, 0.75 USD, 0.50 USD and 0.25 USD respectively, and decreasing the duration of training by 50%.

All costs are reported in 2022 USD and are not discounted, as the BIA focuses on evaluating the actual expected expenditure within a specific budgetary timeframe.

Results

Supplementary Tables 6-9 show the potential budget impact of introducing the E-MOTIVE intervention in public hospitals in the participating countries over a 5-year period. The base case analysis suggests introducing E-MOTIVE in South Africa could decrease costs to the public healthcare system. In Kenya, Nigeria and South Africa, where the calibrated drape is more costly relative to hospital services, a modest increase in budget would be required to achieve substantially improved PPH-related outcomes. However, in the years following the completion of the initial training to deliver the intervention, the budget impact decreases.

Sensitivity analyses suggest that as the cost of the calibrated drape is reduced, feasibly due to expanded production, the E-MOTIVE intervention becomes substantially more affordable.

It should be noted that these potential budget impact estimates were derived from country-level cost analyses, which for pragmatism are based on whole-trial clinical and utilisation data. Although fully country-specific data were not applied, we believe they provide a useful indication of the potential implications of introducing the E-MOTIVE intervention on public healthcare system expenditure. Nevertheless, these estimates should be interpreted with caution.

Supplementary Table 6. Potential budget impact of introducing the E-MOTIVE intervention in Kenya (2022 USD)					
	**Year 1 (2024)**	**Year 2 (2025)**	**Year 3 (2026)**	**Year 4 (2027)**	**Year 5 (2028)**
Total Births	1,519,473	1,540,132	1,558,304	1,581,799	1,596,024
Public Hospital Births	664,010	673,038	680,979	691,246	697,462
Public Hospital Births by Vaginal Delivery	524,568	531,700	537,973	546,084	550,995
**Base-Case**	**Total Cost (2022 USD)**				
Usual Care	7,298,063	7,397,289	7,484,570	7,597,417	7,665,740
E-MOTIVE Introduced	7,398,855	7,914,271	8,268,337	8,185,451	8,259,062
Budget Impact (USD)	100,791	516,982	783,767	588,035	593,323
**Sensitivity Analysis**	**Total Cost (2022 USD)**				
E-MOTIVE Introduced (Training Costs Reduced by 50%)	7,376,702	7,813,230	8,166,103	8,185,451	8,259,062
Budget Impact (USD)	78,639	415,941	681,534	588,035	593,323
E-MOTIVE Introduced (Calibrated Drape Cost - 1 USD)	7,382,909	7,825,380	8,104,809	8,019,458	8,091,576
Budget Impact	84,846	428,091	620,240	422,041	425,837
E-MOTIVE Introduced (Calibrated Drape Cost - 0.75 USD)	7,366,963	7,736,480	7,941,265	7,853,448	7,924,074
Budget Impact	68,899	339,191	456,696	256,032	258,334
E-MOTIVE Introduced (Calibrated Drape Cost - 0.50 USD)	7,351,016	7,647,580	7,777,721	7,687,439	7,756,571
Budget Impact	52,952	250,291	293,152	90,022	90,832
E-MOTIVE Introduced (Calibrated Drape Cost - 0.25 USD)	7,335,069	7,558,679	7,614,178	7,521,429	7,589,069
Budget Impact	37,006	161,390	129,608	-75,988	-76,671
Drape device cost reported is prior to adjustment to 2022 USD and 25% adjustment for shipping and handling.					

Supplementary Table 7. Potential budget impact of introducing the E-MOTIVE intervention in Nigeria (2022 USD)					
	**Year 1 (2024)**	**Year 2 (2025)**	**Year 3 (2026)**	**Year 4 (2027)**	**Year 5 (2028)**
Total Births	8,234,398	8,321,052	8,417,807	8,515,800	8,600,296
Public Hospital Births	1,081,176	1,081,176	1,081,176	1,081,176	1,081,176
Public Hospital Births by Vaginal Delivery	991,439	1,001,872	1,013,522	1,025,320	1,035,494
**Base-Case**	**Total Cost (2022 USD)**				
Usual Care	21,144,336	21,366,847	21,615,295	21,866,922	22,083,891
E-MOTIVE Introduced	21,240,980	21,872,445	22,427,802	22,543,664	22,767,348
Budget Impact	96,643	505,598	812,507	676,742	683,457
**Sensitivity Analysis**	**Total Cost 2022 (USD)**				
E-MOTIVE Introduced (Training Costs Reduced by 50%)	21,225,377	21,801,494	22,356,026	22,543,664	22,767,348
Budget Impact	81,041	434,647	740,731	676,742	683,457
E-MOTIVE Introduced (Calibrated Drape Cost - 1 USD)	21,210,843	21,704,949	22,119,721	22,231,997	22,452,589
Budget Impact	66,507	338,102	504,427	365,076	368,698
E-MOTIVE Introduced (Calibrated Drape Cost - 0.75 USD)	21,180,700	21,537,419	21,811,581	21,920,269	22,137,768
Budget Impact	36,364	170,572	196,286	53,347	53,877
E-MOTIVE Introduced (Calibrated Drape Cost - 0.50 USD)	21,150,560	21,369,906	21,503,470	21,608,572	21,822,978
Budget Impact	6,224	3,059	-111,825	-258,350	-260,913
E-MOTIVE Introduced (Calibrated Drape Cost - 0.25 USD)	21,120,424	21,202,409	21,195,390	21,296,905	21,508,219
Budget Impact	-23,913	-164,437	-419,905	-570,017	-575,672
Drape device cost reported is prior to adjustment to 2022 USD and 25% adjustment for shipping and handling.					

Supplementary Table 8. Potential budget impact of introducing the E-MOTIVE intervention in South Africa (2022 USD)					
	**Year 1 (2024)**	**Year 2 (2025)**	**Year 3 (2026)**	**Year 4 (2027)**	**Year 5 (2028)**
Total Births	1,127,932	1,114,545	1,105,630	1,097,949	1,089,004
Public Hospital Births	774,664	765,470	759,347	754,071	743,319
Public Hospital Births by Vaginal Delivery	579,448	572,571	567,991	564,045	559,450
**Base-Case**	**Total Cost (2022 USD)**				
Usual Care	94,469,103	93,347,884	92,601,215	91,957,899	91,208,717
E-MOTIVE Introduced	94,295,586	92,276,264	90,198,689	89,001,963	88,276,863
Budget Impact	-173,517	-1,071,621	-2,402,526	-2,955,936	-2,931,854
**Sensitivity Analysis**	**Total Cost (2022 USD)**				
E-MOTIVE Introduced (Training Costs Reduced by 50%)	94,230,511	91,986,905	89,911,645	89,001,963	88,276,863
Budget Impact	-238,591	-1,360,980	-2,689,571	-2,955,936	-2,931,854
E-MOTIVE Introduced (Calibrated Drape Cost - 1 USD)	94,277,970	92,180,530	90,026,020	88,830,493	88,106,790
Budget Impact	-191,132	-1,167,354	-2,575,195	-3,127,406	-3,101,927
E-MOTIVE Introduced (Calibrated Drape Cost - 0.75 USD)	94,260,355	92,084,796	89,853,351	88,659,023	87,936,717
Budget Impact	-208,747	-1,263,088	-2,747,864	-3,298,876	-3,272,000
E-MOTIVE Introduced (Calibrated Drape Cost - 0.50 USD)	94,242,740	91,989,062	89,680,681	88,487,553	87,766,644
Budget Impact	-226,363	-1,358,822	-2,920,534	-3,470,346	-3,442,073
E-MOTIVE Introduced (Calibrated Drape Cost - 0.25 USD)	94,225,125	91,893,328	89,508,012	88,316,083	87,596,571
Budget Impact	-243,978	-1,454,556	-3,093,203	-3,641,815	-3,612,146
Drape device cost reported is prior to adjustment to 2022 USD and 25% adjustment for shipping and handling.					

Supplementary Table 9. Potential budget impact of introducing the E-MOTIVE intervention in Tanzania (2022 USD)					
	**Year 1 (2024)**	**Year 2 (2025)**	**Year 3 (2026)**	**Year 4 (2027)**	**Year 5 (2028)**
Total Births	2,416,633	2,446,942	2,482,397	2,517,660	2,554,608
Public Hospital Births	581,925	589,224	597,761	606,253	615,150
Public Hospital Births by Vaginal Delivery	492,891	499,072	506,304	513,496	521,032
**Base-Case**	**Total Cost (2022 USD)**				
Usual Care	5,114,692	5,178,840	5,253,878	5,328,511	5,406,710
E-MOTIVE Introduced	5,192,126	5,594,441	5,962,097	5,974,437	6,062,115
Budget Impact	77,434	415,601	708,219	645,926	655,406
**Sensitivity Analysis**	**Total Cost (2022 USD)**				
E-MOTIVE Introduced (Training Costs Reduced by 50%)	5,184,409	5,559,280	5,926,427	5,974,437	6,062,115
Budget Impact	69,717	380,441	672,549	645,926	655,406
E-MOTIVE Introduced (Calibrated Drape Cost - 1 USD)	5,177,142	5,510,996	5,808,181	5,818,335	5,903,722
Budget Impact	62,450	332,156	554,302	489,824	497,012
E-MOTIVE Introduced (Calibrated Drape Cost - 0.75 USD)	5,162,158	5,427,551	5,654,264	5,662,232	5,745,328
Budget Impact	47,466	248,711	400,386	333,721	338,619
E-MOTIVE Introduced (Calibrated Drape Cost - 0.50 USD)	5,147,174	5,344,106	5,500,348	5,506,129	5,586,935
Budget Impact	32,482	165,266	246,470	177,618	180,225
E-MOTIVE Introduced (Calibrated Drape Cost - 0.25 USD)	5,132,191	5,260,664	5,346,437	5,350,032	5,428,546
Budget Impact	17,499	81,824	92,558	21,521	21,836
Drape device cost reported is prior to adjustment to 2022 USD and 25% adjustment for shipping and handling.					

References

1. United Nations, D.o.E.a.S.A., Population Division. *World Population Prospects 2022, Online Edition.*, (2022).
2. KNBS and ICF. Kenya Demographic and Health Survey 2022 [Dataset]. KEBR8BDT.DTA. (KNBS and ICF [Producers], ICF [Distributor], Nairobi, Kenya, and Rockville, Maryland, USA, 2023).
3. National Population Commission - NPC and ICF. Nigeria Demographic and Health Survey 2018 [Dataset] NGBR7BFL.DTA. (NPC and ICF [Producers]. ICF [Distributor], Abuja, Nigeria, 2019).
4. ICF, N.D.o.H.a. South Africa Demographic and Health Survey 2016 [Dataset]. ZAAH71FL.DTA. (National Department of Health - NDoH - ICF [Producers]. ICF [Distributor], Pretoria, 2019).
5. Ministry of Health, C.D., Gender, Elderly*, et al.* Tanzania Demographic and Health Survey and Malaria Indicator Survey 2015-2016 [Dataset]. TZBR7BDT.DTA. (MoHCDGEC, MoH, NBS, OCGS, and ICF [Producers]. ICF [Distributor], Dar es Salaam, Tanzania, 2016).
