# Supplementary material for: A cost-effectiveness analysis of early detection and bundled treatment of postpartum hemorrhage alongside the E-MOTIVE trial
Source: Nat Med. 2024 Jun 6;30(8):2343–8. doi: 10.1038/s41591-024-03069-5 (PMC11333277; doi:10.1038/s41591-024-03069-5)
Supplement: Supplementary file 2 — Reporting Summary [file 41591_2024_3069_MOESM2_ESM.pdf]

nature portfolio

Corresponding author(s): Prof. Tracy E. Roberts

Last updated by author(s): May 8, 2024

# Reporting Summary

Nature Portfolio wishes to improve the reproducibility of the work that we publish. This form provides structure for consistency and transparency in reporting. For further information on Nature Portfolio policies, see our Editorial Policies and the Editorial Policy Checklist.

## Statistics

For all statistical analyses, confirm that the following items are present in the figure legend, table legend, main text, or Methods section.

| n/a | Confirmed | |
|---|---|---|
| ☐ | ☒ | The exact sample size (*n*) for each experimental group/condition, given as a discrete number and unit of measurement |
| ☐ | ☒ | A statement on whether measurements were taken from distinct samples or whether the same sample was measured repeatedly |
| ☒ | ☐ | The statistical test(s) used AND whether they are one- or two-sided<br>*Only common tests should be described solely by name; describe more complex techniques in the Methods section.* |
| ☐ | ☒ | A description of all covariates tested |
| ☒ | ☐ | A description of any assumptions or corrections, such as tests of normality and adjustment for multiple comparisons |
| ☐ | ☒ | A full description of the statistical parameters including central tendency (e.g. means) or other basic estimates (e.g. regression coefficient) AND variation (e.g. standard deviation) or associated estimates of uncertainty (e.g. confidence intervals) |
| ☐ | ☒ | For null hypothesis testing, the test statistic (e.g. *F*, *t*, *r*) with confidence intervals, effect sizes, degrees of freedom and *P* value noted<br>*Give P values as exact values whenever suitable.* |
| ☒ | ☐ | For Bayesian analysis, information on the choice of priors and Markov chain Monte Carlo settings |
| ☒ | ☐ | For hierarchical and complex designs, identification of the appropriate level for tests and full reporting of outcomes |
| ☒ | ☐ | Estimates of effect sizes (e.g. Cohen's *d*, Pearson's *r*), indicating how they were calculated |

*Our web collection on statistics for biologists contains articles on many of the points above.*

## Software and code

Policy information about availability of computer code

| | |
|---|---|
| Data collection | REDCap version 10.9.0-13.3.2 |
| Data analysis | Stata version 17.1 |

For manuscripts utilizing custom algorithms or software that are central to the research but not yet described in published literature, software must be made available to editors and reviewers. We strongly encourage code deposition in a community repository (e.g. GitHub). See the Nature Portfolio guidelines for submitting code & software for further information.

## Data

Policy information about availability of data

All manuscripts must include a data availability statement. This statement should provide the following information, where applicable:

- Accession codes, unique identifiers, or web links for publicly available datasets
- A description of any restrictions on data availability
- For clinical datasets or third party data, please ensure that the statement adheres to our policy

Patient data cannot be made publicly available due to privacy concerns. The complete de-identified patient data that support the findings of this study can be obtained from the Chief Investigator of the E-MOTIVE trial, on approval from the E-MOTIVE Trial Data Analysis Sub-Committee. Approval from this committee can be requested by directly contacting the Chief Investigator (a.coomarasamy@bham.ac.uk), with an expected review period of approximately 2–3 months. After approval, researchers will be granted access to perform analyses, ensuring data security and confidentiality, with measures in place to prevent any breach of

personal information. Additional data used for the analysis are publicly available and referenced in the Methods and Supplementary Information. The parameter values and their sources are reported in Extended Data Table 2 and Supplementary Tables 6-9.Patient data cannot be made publicly available due to privacy concerns. The complete de-identified patient data that support the findings of this study can be obtained from the Chief Investigator of the E-MOTIVE trial, on approval from the E-MOTIVE Trial Data Analysis Sub-Committee. Approval from this committee can be requested by directly contacting the Chief Investigator (a.coomarasamy@bham.ac.uk), with an expected review period of approximately 2–3months. After approval, researchers will be granted access to perform analyses, ensuring data security and confidentiality, with measures in place to prevent any breach of personal information. Additional data used for the analysis are publicly available and referenced in the Methods and Supplementary Information. The parameter values and their sources are reported in Extended Data Table 2 and Supplementary Tables 6-9. Stata codes are available at https://github.com/ewbham/E-MOTIVE

## Research involving human participants, their data, or biological material

Policy information about studies with human participants or human data. See also policy information about sex, gender (identity/presentation), and sexual orientation and race, ethnicity and racism.

**Reporting on sex and gender**

Findings apply to females; study population was female patients having verified vaginal birth in the study facilities

**Reporting on race, ethnicity, or other socially relevant groupings**

Covariates regarding race, ethnicity or other socially relevant groupings were not collected or used at any stage in the analysis.

**Population characteristics**

Covariate-relevant characteristics (age, parity, past and current diagnosis) were collected as part of the clinical trial dataset but were not used in the present study.

**Recruitment**

Hospitals were eligible for inclusion if they were geographically and administratively distinct from each other, had between 1000 and 5000 vaginal births per year, and were able to provide comprehensive obstetrical care with the ability to perform surgery for PPH. Hospitals were excluded if they had already implemented a treatment bundle for PPH. Written permission was granted by each participating hospital for clinical staff to extract anonymised clinical-outcome data for each vaginal birth.

**Ethics oversight**

Ethical approval was granted by the University of Birmingham Science, Technology, Engineering and Mathematics (STEM) ethics committee in the UK; the World Health Organization - Human Reproduction Programme (WHO-HRP) (approval for formative phase) in Switzerland; the Kenyatta National Hospital (KNH) - University of Nairobi (UoN) Ethics and Research Committee, the National Commission for Science, Technology and Innovation (NACOSTI), and the Pharmacy and Poisons Board (PPB) in Kenya; the National Health Research Ethics Committee of Nigeria (NHREC) and National Agency for Food and Drug Administration and Control (NAFDAC) in Nigeria; the University of the Witwatersrand Human Research Ethics Committee (Medical), the Eastern Cape Department of Health - Eastern Cape Health Research Committee, the KwaZulu-Natal Department of Health - KZN Health Research Committee, and the University of Cape Town - Human Research Ethics Committee in South Africa; the Muhimbili University of Health and Allied Sciences (MUHAS) - Senate Research and Publications Committee, and the National Institute for Medical Research in Tanzania.

Note that full information on the approval of the study protocol must also be provided in the manuscript.

# Field-specific reporting

Please select the one below that is the best fit for your research. If you are not sure, read the appropriate sections before making your selection.

☐ Life sciences ☒ Behavioural & social sciences ☐ Ecological, evolutionary & environmental sciences

For a reference copy of the document with all sections, see nature.com/documents/nr-reporting-summary-flat.pdf

# Behavioural & social sciences study design

All studies must disclose on these points even when the disclosure is negative.

**Study description**

A trial-based cost-effectiveness analysis to determine whether the E-MOTIVE intervention, which included a calibrated blood-collection drape for early detection of PPH and a bundle of first-response treatments (uterine massage, oxytocic drugs, tranexamic acid, intravenous fluids, examination, and escalation), was cost-effective compared with usual care.

**Research sample**

Females of reproductive age in Kenya, Nigeria, South Africa and Tanzania who gave birth in secondary level hospitals included in the E-MOTIVE clinical trial. The 78 hospitals (14 in Kenya, 38 in Nigeria, 14 in South Africa, and 12 in Tanzania) included in the cluster randomised trial were representative of the target population. Median age in both arms was similar 26 (21–31) in the intervention group and 26 (21–30) in the usual care group. The present analysis was designed as an economic evaluation of the E-MOTIVE trial. Detail on justification for the trial's research sample can be found in the clinical paper.

**Sampling strategy**

Cluster-randomised trial. The sample size calculation was made based on the assumption that there were 80 health facilities in the trial, evenly split across the intervention and control groups, with an average number of 192 births per health facility per month. The anticipated total sample size for the study (running for 14 months) would be 215,040 (=8019214). The number of health facilities (80) was inflated by 10% to allow for dropout from the number of health facilities required (72). Calculations on expected levels of power indicated that the study would have at least 90% power at 5% significance (two-sided) to detect a 30% RRR for most scenarios after allowing for clustering and for varying cluster size. The study would have over 90% power to detect smaller RRR if the ICC is close to

| | |
|---|---|
| | the lower bound (0.001), the CAC is at the upper bound (1.0), or the prevalence of the study is relatively large (4.0%). |
| Data collection | Data on blood loss were source-verified by capturing a photograph of a blood-collection drape with collected blood inside it, positioned on a digital weighing scale, with the weight visible in the photograph. Clinical trial data were collected using case report forms. Blinding was not possible given the nature of the intervention and cluster trial design. |
| Timing | Between August and October 2021, participating hospitals entered a 7-month baseline period. After this 7-month baseline period, hospitals were randomly assigned in a sequential manner as they approached the end of their assigned baseline phase either to continue providing usual care or to receive the trial intervention for 7 months, with an allowance of 2 months for transition period. |
| Data exclusions | Patients with missing verified blood loss data were excluded from the primary economic analysis (1704 in the intervention group and 1972 in the usual care group) - 2% of patients. Their data are reflected in the sensitivity analysis using multiple imputation |
| Non-participation | Two hospitals, 1 in each trial group, did not receive the assigned intervention because of participation in a conflicting program and were not included in the analyses. |
| Randomization | Cluster-randomised trial. Randomisation was implemented using a minimisation algorithm to ensure a balance of the intervention and control facilities for the following (measured at the cluster-level during the first 5 months of the baseline phase): 1. Number of vaginal births 2. Proportion of births with the composite primary outcome (before randomisation) 3. Oxytocin quality 4. Number of intervention and control clusters in each country. Hospitals were randomised in a 1:1 ratio. |

# Reporting for specific materials, systems and methods

We require information from authors about some types of materials, experimental systems and methods used in many studies. Here, indicate whether each material, system or method listed is relevant to your study. If you are not sure if a list item applies to your research, read the appropriate section before selecting a response.

## Materials & experimental systems

| n/a | Involved in the study |
|---|---|
| ☒ | ☐ Antibodies |
| ☒ | ☐ Eukaryotic cell lines |
| ☒ | ☐ Palaeontology and archaeology |
| ☒ | ☐ Animals and other organisms |
| ☐ | ☒ Clinical data |
| ☒ | ☐ Dual use research of concern |
| ☒ | ☐ Plants |

## Methods

| n/a | Involved in the study |
|---|---|
| ☒ | ☐ ChIP-seq |
| ☒ | ☐ Flow cytometry |
| ☒ | ☐ MRI-based neuroimaging |

# Clinical data

Policy information about clinical studies

All manuscripts should comply with the ICMJE guidelines for publication of clinical research and a completed CONSORT checklist must be included with all submissions.

| | |
|---|---|
| Clinical trial registration | Trial registration number: NCT04341662 |
| Study protocol | The trial protocol can be accessed at https://www.birmingham.ac.uk/research/bctu/trials/womens/emotive/e-motive |
| Data collection | Data were collected at secondary-level hospitals in Kenya, Nigeria, South Africa and Tanzania. Data collection began between August and October 2021 and lasted for 16 months. |
| Outcomes | Severe PPH, Costs and DALYs are equivalent to the primary outcomes. Severe PPH (blood loss >= 1000ml) (source-verified by capturing a photograph of a blood-collection drape with collected blood inside it, positioned on a digital weighing scale, with the weight visible in the photograph) was the primary clinical outcome used in the economic analysis. Costs and disability-adjusted life-years were derived from resource use outcomes (i.e. duration of hospitalisation, use of tranexamic acid, blood transfusions) and clinical outcomes (death from bleeding, PPH, severe PPH) within the dataset. This study did not include secondary outcomes. |

## Plants

| | |
|---|---|
| Seed stocks | *Report on the source of all seed stocks or other plant material used. If applicable, state the seed stock centre and catalogue number. If plant specimens were collected from the field, describe the collection location, date and sampling procedures.* |
| Novel plant genotypes | *Describe the methods by which all novel plant genotypes were produced. This includes those generated by transgenic approaches, gene editing, chemical/radiation-based mutagenesis and hybridization. For transgenic lines, describe the transformation method, the number of independent lines analyzed and the generation upon which experiments were performed. For gene-edited lines, describe the editor used, the endogenous sequence targeted for editing, the targeting guide RNA sequence (if applicable) and how the editor was applied.* |
| Authentication | *Describe any authentication procedures for each seed stock used or novel genotype generated. Describe any experiments used to assess the effect of a mutation and, where applicable, how potential secondary effects (e.g. second site T-DNA insertions, mosiacism, off-target gene editing) were examined.* |
